# Foliar Phenotypic Plasticity Reflects Adaptation to Environmental Variability

**DOI:** 10.3390/plants12102041

**Published:** 2023-05-19

**Authors:** William W. Adams, Jared J. Stewart, Stephanie K. Polutchko, Christopher M. Cohu, Onno Muller, Barbara Demmig-Adams

**Affiliations:** 1 Department of Ecology and Evolutionary Biology, University of Colorado, Boulder, CO 80309-0334, USA; jared.stewart@colorado.edu (J.J.S.); stephanie.polutchko@colorado.edu (S.K.P.); barbara.demmig-adams@colorado.edu (B.D.-A.); 2 Environmental Science and Technology, Colorado Mesa University, Grand Junction, CO 81502, USA; ccohu@coloradomesa.edu; 3 Pflanzenwissenschaften (IBG-2), Institut für Bio- und Geowissenschaften, Forschungszentrum Jülich, 52428 Jülich, Germany; o.muller@fz-juelich.de

**Keywords:** foliar vasculature, leaf venation, light acclimation, phloem, photosynthesis, precipitation cline, temperature acclimation, temperature cline, transpiration, xylem

## Abstract

*Arabidopsis thaliana* ecotypes adapted to native habitats with different daylengths, temperatures, and precipitation were grown experimentally under seven combinations of light intensity and leaf temperature to assess their acclimatory phenotypic plasticity in foliar structure and function. There were no differences among ecotypes when plants developed under moderate conditions of 400 µmol photons m^−2^ s^−1^ and 25 °C. However, in response to more extreme light or temperature regimes, ecotypes that evolved in habitats with pronounced differences in either the magnitude of changes in daylength or temperature or in precipitation level exhibited pronounced adjustments in photosynthesis and transpiration, as well as anatomical traits supporting these functions. Specifically, when grown under extremes of light intensity (100 versus 1000 µmol photons m^−2^ s^−1^) or temperature (8 °C versus 35 °C), ecotypes from sites with the greatest range of daylengths and temperature over the growing season exhibited the greatest differences in functional and structural features related to photosynthesis (light- and CO_2_-saturated capacity of oxygen evolution, leaf dry mass per area or thickness, phloem cells per minor vein, and water-use efficiency of CO_2_ uptake). On the other hand, the ecotype from the habitat with the lowest precipitation showed the greatest plasticity in features related to water transport and loss (vein density, ratio of water to sugar conduits in foliar minor veins, and transpiration rate). Despite these differences, common structure–function relationships existed across all ecotypes and growth conditions, with significant positive, linear correlations (i) between photosynthetic capacity (ranging from 10 to 110 µmol O_2_ m^−2^ s^−1^) and leaf dry mass per area (from 10 to 75 g m^−2^), leaf thickness (from 170 to 500 µm), and carbohydrate-export infrastructure (from 6 to 14 sieve elements per minor vein, from 2.5 to 8 µm^2^ cross-sectional area per sieve element, and from 16 to 82 µm^2^ cross-sectional area of sieve elements per minor vein); (ii) between transpiration rate (from 1 to 17 mmol H_2_O m^−2^ s^−1^) and water-transport infrastructure (from 3.5 to 8 tracheary elements per minor vein, from 13.5 to 28 µm^2^ cross-sectional area per tracheary element, and from 55 to 200 µm^2^ cross-sectional area of tracheary elements per minor vein); (iii) between the ratio of transpirational water loss to CO_2_ fixation (from 0.2 to 0.7 mol H_2_O to mmol^−1^ CO_2_) and the ratio of water to sugar conduits in minor veins (from 0.4 to 1.1 tracheary to sieve elements, from 4 to 6 µm^2^ cross-sectional area of tracheary to sieve elements, and from 2 to 6 µm^2^ cross-sectional area of tracheary elements to sieve elements per minor vein); (iv) between sugar conduits and sugar-loading cells; and (v) between water conducting and sugar conducting cells. Additionally, the proportion of water conduits to sugar conduits was greater for all ecotypes grown experimentally under warm-to-hot versus cold temperature. Thus, developmental acclimation to the growth environment included ecotype-dependent foliar structural and functional adjustments resulting in multiple common structural and functional relationships.

## 1. Introduction

Plant phenotypic plasticity (acclimatory adjustment in response to abiotic or biotic features of an individual plant’s growth environment) in structure, function, phenology, or defense against herbivores and pathogens has been the subject of numerous studies (for reviews, see [1,2,3,4,5,6,7,8,9,10,11]). De Kroon et al. [5] described plant phenotypic plasticity displayed at the level of individual organs, and Niklas [12] illustrated plasticity both within and among individual plant cells. For example, different portions of the root system respond individually to unevenly distributed nutrients [13], and leaves acclimate differentially in sun-exposed versus shaded portions of a single plant [14,15,16,17,18].

**Figure 1:**
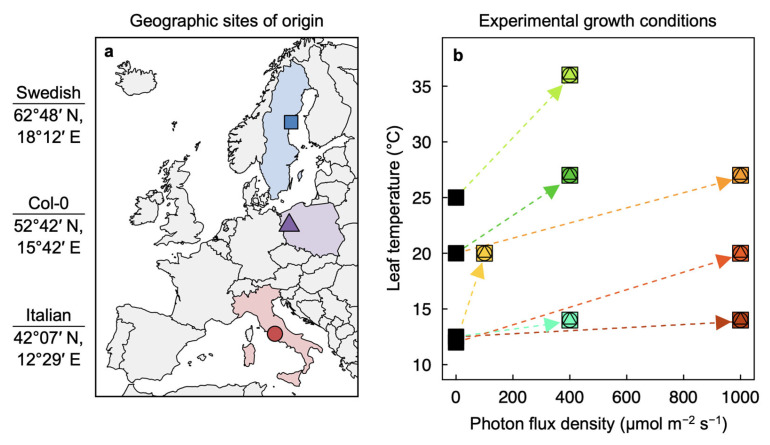
(**a**) Locations and coordinates from which the three ecotypes of *Arabidopsis thaliana* characterized in this study originated and (**b**) seven temperature and light regimes (9-h photoperiod) under which plants were experimentally grown. Squares (Swedish ecotype), triangles (Col-0 wild type, or Polish ecotype), and circles (Italian ecotype) with these colors (designating each of the experimental growth regimes) are used in Figures 2 and 4–11. In (**b**), the black squares at 0 µmol photons m^−2^ s^−1^ represent air and leaf temperatures during the 15-h dark period with dashed lines showing the change in temperature and photon flux density (PFD) from the dark period to the photoperiod. Air temperatures during growth were (i) 12.5 °C during the night and 8 °C during the day to result in a leaf temperature of 14 °C during the photoperiod (under either moderate (400 µmol photons m^−2^ s^−1^) or high (1000 µmol photons m^−2^ s^−1^) PFD); (ii) 20 °C during the night and 25 °C during the day to result in a leaf temperature of 27 °C during the photoperiod (under either moderate or high light); (iii) 12 °C during the night and 20 °C during the day to result in a leaf temperature of 20 °C under low PFD (100 µmol photons m^−2^ s^−1^); (iv) 12 °C during the night and day to result in a leaf temperature of 20 °C under high PFD; and (v) 25 °C during the night and 35 °C during the day to result in a leaf temperature of 36 °C under moderate PFD. For additional details regarding (**a**), see Ågren and Schemske [21] and Adams et al. [22]. For additional details regarding (**b**), see Cohu et al. [23,24], Adams et al. [22,25], and Stewart et al. [26,27,28].

The extent of phenotypic plasticity has been compared among ecotypes adapted to differing conditions over a species’ geographic range using common-garden and reciprocal-transplant experiments. *Arabidopsis thaliana* ecotypes from multiple sites of origin exhibited no differences in survival or reproductive fitness when grown at the southernmost site of origin of these ecotypes [19]. In another common-garden *A. thaliana* experiment [20], the best predictor of plant performance was the latitude (and corresponding climate) from which each ecotype originated. Reciprocal transplant experiments over five years with the Swedish and Italian ecotypes employed in the present study (at the extremes of a latitudinal transect; Figure 1a) repeatedly demonstrated greater reproductive fitness of the native over the transplanted ecotype, as well as significantly greater survival of each, in its respective habitat [21].

One major focus of studies on the latter two ecotypes has been the greater freezing tolerance of the Swedish compared to the Italian ecotype and the underlying differences in regulons responsible for that difference [29,30,31,32,33]. In the present study, traits related to plant performance at above-freezing temperatures are examined in these two as well as a third ecotype from a site in Poland (Columbia-0 accession—the standard wild-type of this model plant) at almost an equal distance between the other two ecotypes’ sites of origin (Figure 1a). The leaf, as the primary site of photosynthesis that underpins most terrestrial life [34,35], has received considerable attention regarding its plasticity [36]. Nevertheless, relationships between photosynthesis-associated leaf traits and the plant-growth environment have remained elusive (see [37]). In the present study, we examined leaves’ well-characterized acclimatory adjustments to light intensity (photon flux density, PFD) and temperature [25,38,39,40,41,42,43,44,45,46,47,48,49,50,51] for ecotypes of *A. thaliana* in the context of variation in these and other environmental factors in each ecotype’s site of origin. We specifically characterized the anatomical and functional traits related to maximal photosynthetic capacity determined from light- and CO_2_-saturated capacity of oxygen evolution. Winter annuals (such as *A. thaliana*), but not summer annuals [50], upregulate this photosynthetic capacity when grown at cool temperatures to a point that allows plants to maintain a similar photosynthesis rate despite the depression of enzyme activity by lower temperature [23,52].

**Figure 2:**
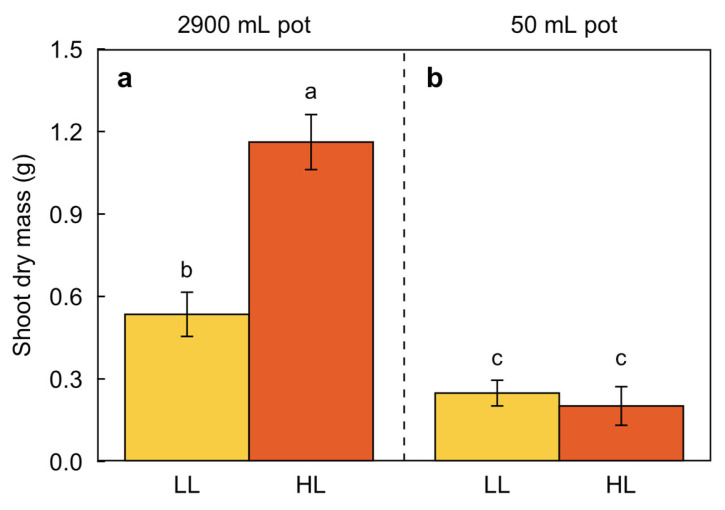
Impact of rooting volume on shoot dry mass of *A. thaliana* (Col-0; originating in Poland—see Figure 1a) grown in (**a**) large pots with 2900 mL volume or (**b**) small pots with 50 mL volume for approximately eight weeks in low light (LL = 100 µmol photons m^−2^ s^−1^; yellow columns) or approximately six weeks in high light (HL = 1000 µmol photons m^−2^ s^−1^; orange columns) under a leaf temperature regime of 20 °C during the 9-h photoperiod and 12 °C during the 15-h dark period (see Figure 1b). Mean values ± standard deviations (*n* = 3 or 4). Significant differences (*p* < 0.05) are indicated by different lower-case letters. Data from Adams et al. [53].

Photosynthesis is regulated not only by environmental inputs such as light but also by the demand for carbohydrates by plant sink tissue [53]. To assess the maximal degree of phenotypic plasticity in response to growth PFD and temperature, we took steps to remove limitations to carbohydrate demand from plant sinks (sink limitation) by other factors via providing ample water, nutrients, and rooting volume. The latter is important, as Nagel et al. [54] showed that roots of *A. thaliana* can grow to a depth of at least 0.3 m, far exceeding the depth of pots typically used to grow this species. Figure 2 demonstrates the effect of rooting volume on shoot dry mass for the widely used *A. thaliana* Columbia-0 accession. A large rooting volume of 2900 mL was used for unlimited growth, with a dramatic effect of increasing the shoot dry mass in both low and especially high growth PFDs (Figure 2a). In contrast, a pot size of 50 mL limited shoot growth under low PFD and prevented high PFD from further stimulating shoot growth (Figure 2b; see Adams et al. [53] for corresponding differences in the maximal photosynthetic capacity as well as images of these plants). In the present study, large pots of 2.9 L were thus used for all experiments. Moreover, whereas leaves of plants growing in small pots were deep red in high light (indicative of sink limitation and oxidative stress), leaf laminae of larger plants in large pots were green with no sign of red even when growing in high light and low temperature (see plant images in [26,27,53]).

**Figure 3:**
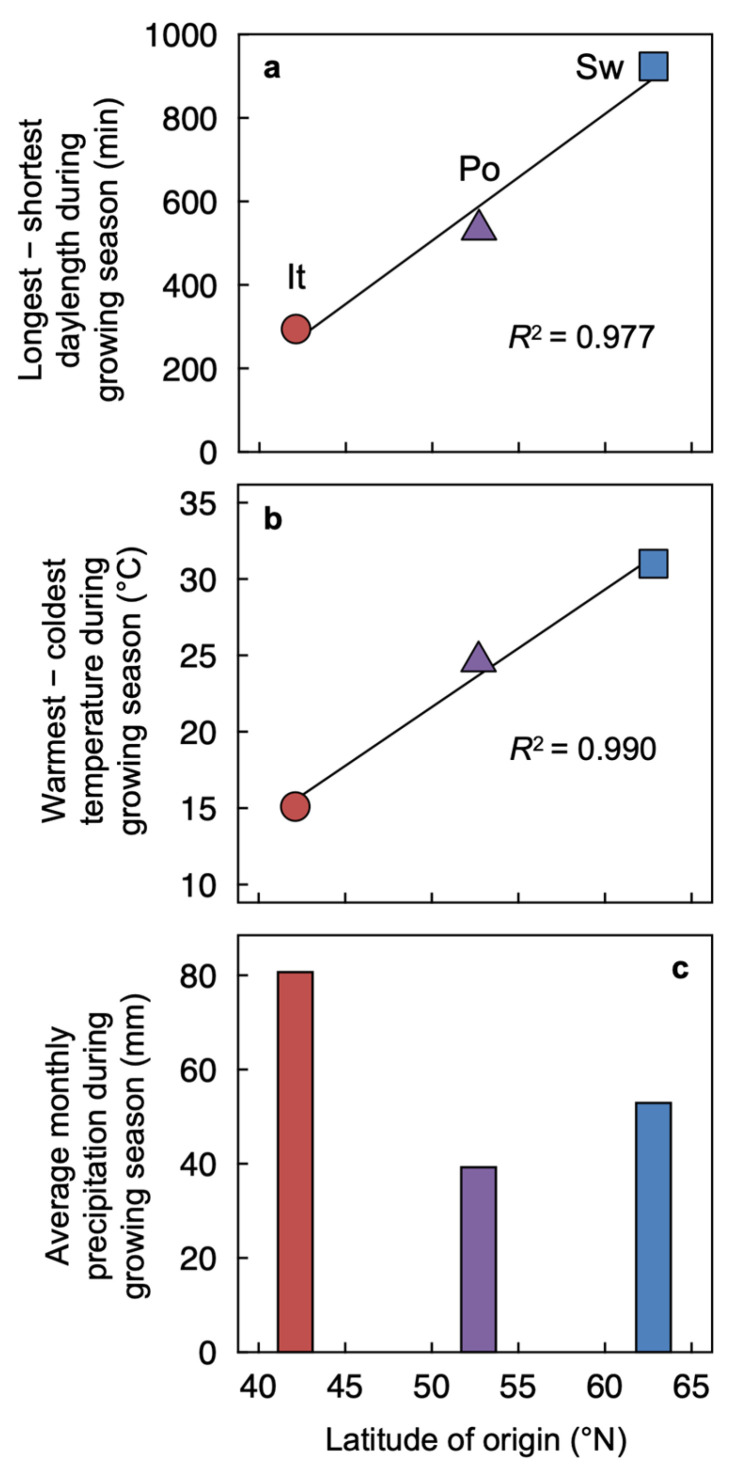
Differences in daylength between the longest and shortest days (**a**) and average monthly maximum and minimum temperatures (**b**), as well as average monthly precipitation (**c**), during the growing season for sites from which each of the three ecotypes originated. The differences were between daylengths and maximum temperatures in April (for the Italian ecotype = It; brick red circles and column), May (for the Polish ecotype = Po; purple triangles and column), and June (for the Swedish ecotype = Sw; blue squares and column), and either 21 December (**a**) or the month of January (**b**). See Figure 2 in Adams et al. [22] for annual variation in daylength and monthly maximum and minimum average temperatures at each of the three sites.

Our specific goal was to relate the extent of phenotypic plasticity of several *A. thaliana* ecotypes under contrasting experimental conditions to the extent of variation in corresponding abiotic conditions across the growing season in the respective ecotype habitats. A comprehensive profile of foliar phenotypic plasticity was established by combining responses of *A. thaliana* ecotypes from habitats that differ in environmental conditions (Col-0 from Poland as well as ecotypes from Sweden and Italy spanning a latitudinal gradient of over 2000 km; Figure 1a) from plants grown experimentally under seven different combinations of leaf temperature and PFD (Figure 1b). The impact of temperature independent of PFD, and vice versa, on foliar acclimation was assessed by comparing plants grown under low versus high temperature under a moderate PFD (blue-green versus olive-green symbols under 400 µmol photons m^−2^ s^−1^ in Figure 1b) and low versus high PFD at a moderate temperature (yellow versus orange symbols at 20 °C in Figure 1b). This impact of the growth regime was evaluated for relationships between foliar structural and functional traits and the climatic conditions experienced by each ecotype during its growing season, specifically differences in daylength (longest—shortest; Figure 3a) or temperature (warmest—coldest; Figure 3b) and average monthly precipitation during the growing season in the respective habitats of origin (Figure 3c). The considerable range of variation in leaf structural and functional traits among the three ecotypes induced by the seven growth regimes also permitted the identification of relationships among foliar parameters that applied across the entire data set.

A number of these traits were previously reported as a function of the latitude of ecotype origin [22,25]. In the current assessment, we asked instead whether the extent of phenotypic plasticity of these traits (in plants grown under different experimental conditions) could be predicted by the extent of variation in associated abiotic conditions in the habitats from which each ecotype originated. Specifically, we compared trait variation with variation in (maximal minus minimal) temperature and/or (maximal minus minimal) daylength across the growing season in the respective habits of origin. We focused on comparing (i) the response of traits related to photosynthetic capacity with vascular sugar-transport infrastructure and (ii) transpiration with vascular water-transport infrastructure. An additional new question addressed whether extreme common experimental growth conditions produced greater differences among ecotypes than moderate common growth conditions. Moreover, the use of seven different combinations of experimental PFD and temperature conditions allowed the assessment of whether any common (significant, linear) relationships exist between (1) photosynthetic capacity and leaf morphological or anatomical traits, (2) transpiration/transpiration ratio and anatomical traits, and (3) different minor vein vascular cell types across all three ecotypes. This comprehensive evaluation was made possible through a combination of previously unpublished data with data from multiple previous studies [22,23,24,25,26,27,28,55].

## 2. Results

### 2.1. Responses to Experimental Conditions as a Function of Environmental Variation in Ecotype Habitat

**Figure 4:**
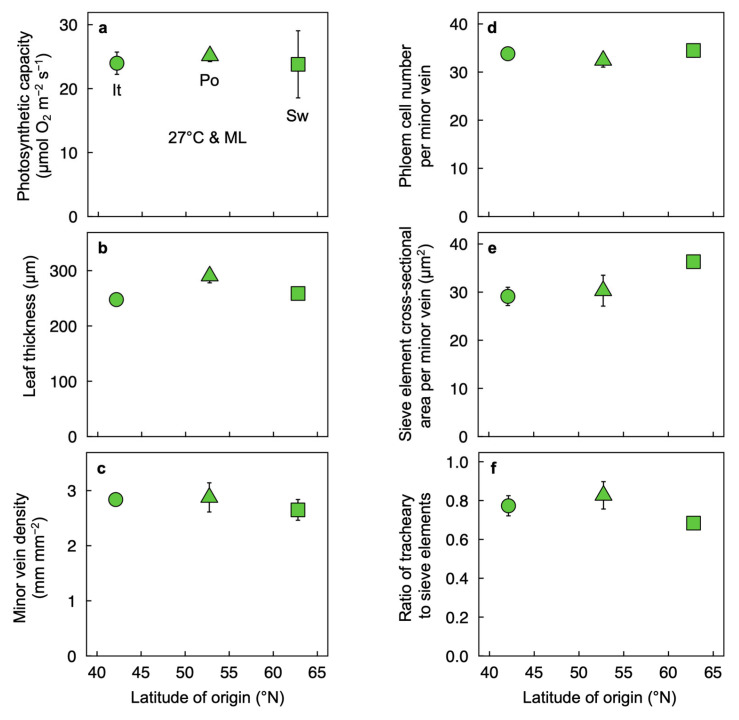
(**a**) Light- and CO_2_-saturated rate of oxygen evolution determined at 25 °C, (**b**) leaf thickness, (**c**) minor vein density (as vein length per unit leaf area), (**d**) number of phloem cells per minor vein, (**e**) cross-sectional area of sieve elements per minor vein, and (**f**) ratio of tracheary to sieve elements in minor veins of *Arabidopsis thaliana* ecotypes from Italy (It, circles), Poland (Po, triangles), and Sweden (Sw, squares) as a function of the latitude from which each originated. Leaves developed under growth conditions of 25 °C (27 °C leaf temperature) and 400 µmol photons m^−2^ s^−1^ (ML) during the 9-h photoperiod and 20 °C during the night. Mean values ± standard deviations (**a**,**c**) or ±standard errors (**b**,**d**–**f**). There were no significant differences among the three ecotypes for any of the parameters except for leaf thickness, which was significantly greater in the Polish compared to the Italian ecotype (*p* < 0.05). For additional information, see Materials and Methods. Data from Cohu et al. [23] and Adams et al. [22].

There was little to no variation in functional or structural leaf traits characterized here among the three ecotypes grown under moderate conditions of 25 °C air temperature and PFD of 400 µmol photons m^−2^ s^−1^ (Figure 4). Whereas leaf thickness was somewhat greater in the Polish compared to the Italian ecotype (Figure 4b), no significant differences in photosynthetic capacity (Figure 4a) or multiple minor-vein features (Figure 4c–f) were seen among the three ecotypes grown under these moderate conditions.

**Figure 5:**
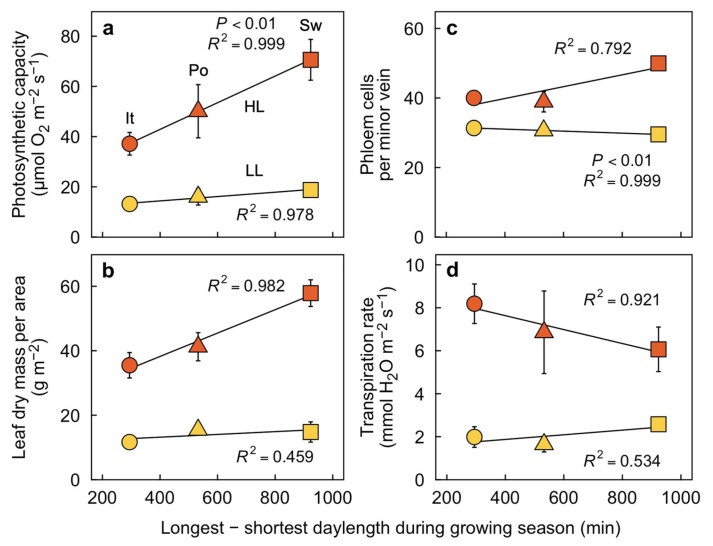
(**a**) Light- and CO_2_-saturated rate of photosynthetic oxygen evolution determined at 25 °C, (**b**) leaf dry mass per area, (**c**) number of phloem cells per minor vein, and (**d**) rate of transpirational water loss (determined under the respective low or high growth PFD, a leaf temperature of 27.0 ± 1.0 °C (*n* = 24), and a vapor pressure deficit of 2.03 ± 0.20 kPa (*n* = 24)) for leaves of *Arabidopsis thaliana* ecotypes from Italy (It, circles), Poland (Po, triangles), and Sweden (Sw, squares) that developed under a 9-h photoperiod of 100 (LL, light orange) or 1000 (HL, orange) µmol photons m^−2^ s^−1^ and a leaf temperature of 20°/12 °C (day/night) as a function of the difference between the longest and shortest days during the growing season for each ecotype in its habitat of origin. Mean values ± standard deviations (**a**,**b**,**d**) or ± standard errors (**c**). For additional information, see Materials and Methods. Data from Stewart et al. [27,28] and Adams et al. [25].

**Figure 6:**
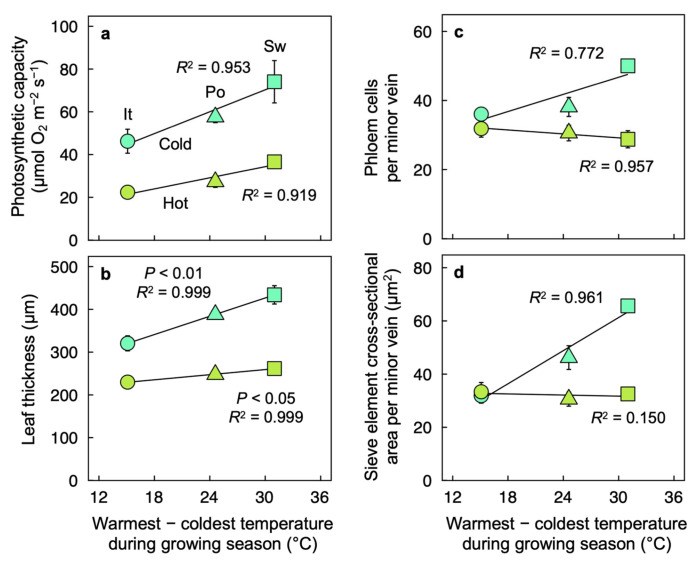
(**a**) Light- and CO_2_-saturated rate of photosynthetic oxygen evolution determined at 25 °C, (**b**) leaf thickness, (**c**) number of phloem cells per minor vein, and (**d**) cross-sectional area of sieve elements comprising the minor veins for leaves of *Arabidopsis thaliana* ecotypes from Italy (It, circles), Poland (Po, triangles), and Sweden (Sw, squares) that developed under 400 µmol photons m^−2^ s^−1^ and a leaf temperature 14 °C (cold, blue-green symbols) or 36 °C (hot, olive-green symbols) during the 9-h photoperiod as a function of the difference between the warmest and coldest temperature during the growing season for each ecotype in its habitat of origin. Mean values ± standard deviations (**a**) or ± standard errors (**b**–**d**). See the legend of Figure 1 and Materials and Methods for additional information. Data from Adams et al. [22] and Stewart et al. [26].

**Figure 7:**
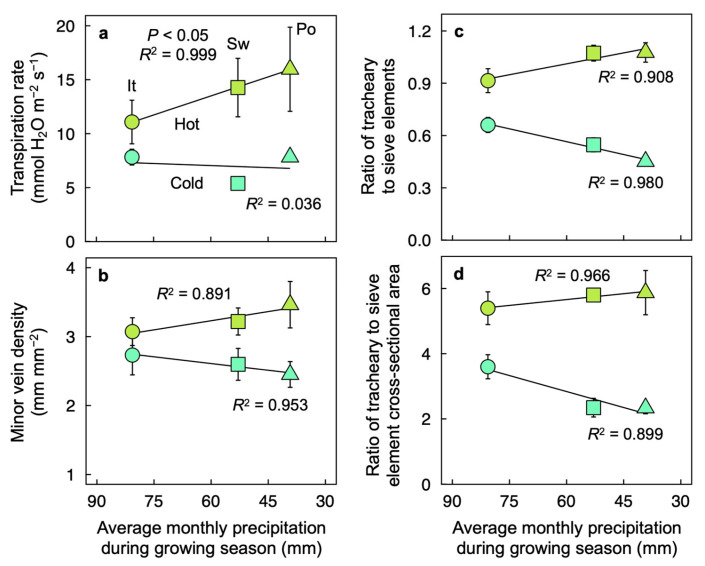
(**a**) Rate of transpirational water loss (determined under 400 µmol photons m^−2^ s^−1^, a leaf temperature of 28.7 ± 1.6 °C (*n* = 29), and vapor pressure deficit of 2.74 ± 0.07 kPa (*n* = 29)), (**b**) minor vein density, (**c**) minor vein ratio of tracheary to sieve elements, and (**d**) ratio of minor vein tracheary to sieve element cross-sectional area for leaves of *Arabidopsis thaliana* ecotypes from Italy (It, circles), Sweden (Sw, squares), and Poland (Po, triangles) that developed under a 9-h photoperiod of 400 µmol photons m^−2^ s^−1^ and a leaf temperature of 36 °C (hot, olive-green symbols) or 14 °C (cold, blue-green symbols) during the photoperiod as a function of average monthly precipitation during the growing season for each ecotype in its habitat of origin. Mean values ± standard deviations (**a**,**b**) or ± standard errors (**c**,**d**). See legend of Figure 1 and Materials and Methods for additional information. Data from Adams et al. [22].

In contrast, plants grown either under two extremes of PFD at a moderate temperature (Figure 5) or two temperature extremes under a moderate PFD (Figure 6 and Figure 7) exhibited strong linear relationships for leaf functional and structural features as a function of the maximal extent of variation in related environmental features (longest—shortest daylength in Figure 5; warmest—coldest temperature in Figure 6) at ecotype site of origin. Firstly, the capacity for light- and CO_2_-saturated photosynthetic oxygen evolution (Figure 5a), leaf dry mass per area (Figure 5b), number of phloem cells per foliar minor vein (Figure 5c), and transpiration rate (Figure 5d) were all higher in leaves grown under high versus low PFD. Furthermore, photosynthetic capacity, leaf dry mass per area, and number of phloem cells per minor vein in plants grown under high PFD (Figure 5a–c) all increased with increasing extent of variation in the daylength over the growing season, i.e., from lowest (Italian ecotype) to highest (Swedish ecotype). On the other hand, the transpiration rate for leaves grown and measured under high PFD declined with increasing habitat daylength amplitude (Figure 5d). It should be noted that the greatest difference between the longest and shortest day occurred in the Swedish site of origin that also features the coldest temperatures (which may impact transpiration dynamics).

Photosynthetic capacity (Figure 6a), leaf thickness (Figure 6b), number of phloem cells per minor vein (Figure 6c), and cross-sectional area of sieve elements per minor vein (Figure 6d) exhibited strong upregulation under cold versus hot temperature (over 100% greater for photosynthetic capacity and sieve element cross-sectional area in the Swedish ecotype), except in the Italian ecotype for the case of phloem-cell or sieve-element cross-sectional area per minor vein (Figure 6c,d). This constitutes a trend of increasing cool-temperature-stimulated upregulation of photosynthetic capacity (and leaf thickness as well as phloem-cell and sieve-element cross-sectional areas per minor vein) with an increasing temperature amplitude (warmest–coldest temperature over the growing season) in the sites of ecotype origin along the transect from Italy to Sweden (Figure 6).

Foliar features related to water transport and loss exhibited linear relationships with precipitation level at the sites of ecotype origin (Figure 7). The transpiration rate (Figure 7a), minor-vein density (Figure 7b), and minor-vein ratios of tracheary-to-sieve elements (Figure 7c) or tracheary-element to sieve-element cross-sectional area (Figure 7d) all exhibited upregulation in plants experimentally grown under high versus low temperature. Furthermore, each of the latter features exhibited a positive linear relationship with precipitation level at the site of origin in plants grown under high temperature but an inverse linear relationship in plants grown under low temperature (Figure 7). These same foliar features exhibited no significant relationships with variation in daylengths nor the temperature range over the growing season in the sites of ecotype origin (not shown).

### 2.2. Magnitude of Response to Growth Environment as a Function of Magnitude of Environmental Variation over the Growing Season in Ecotype Site of Origin

**Figure 8:**
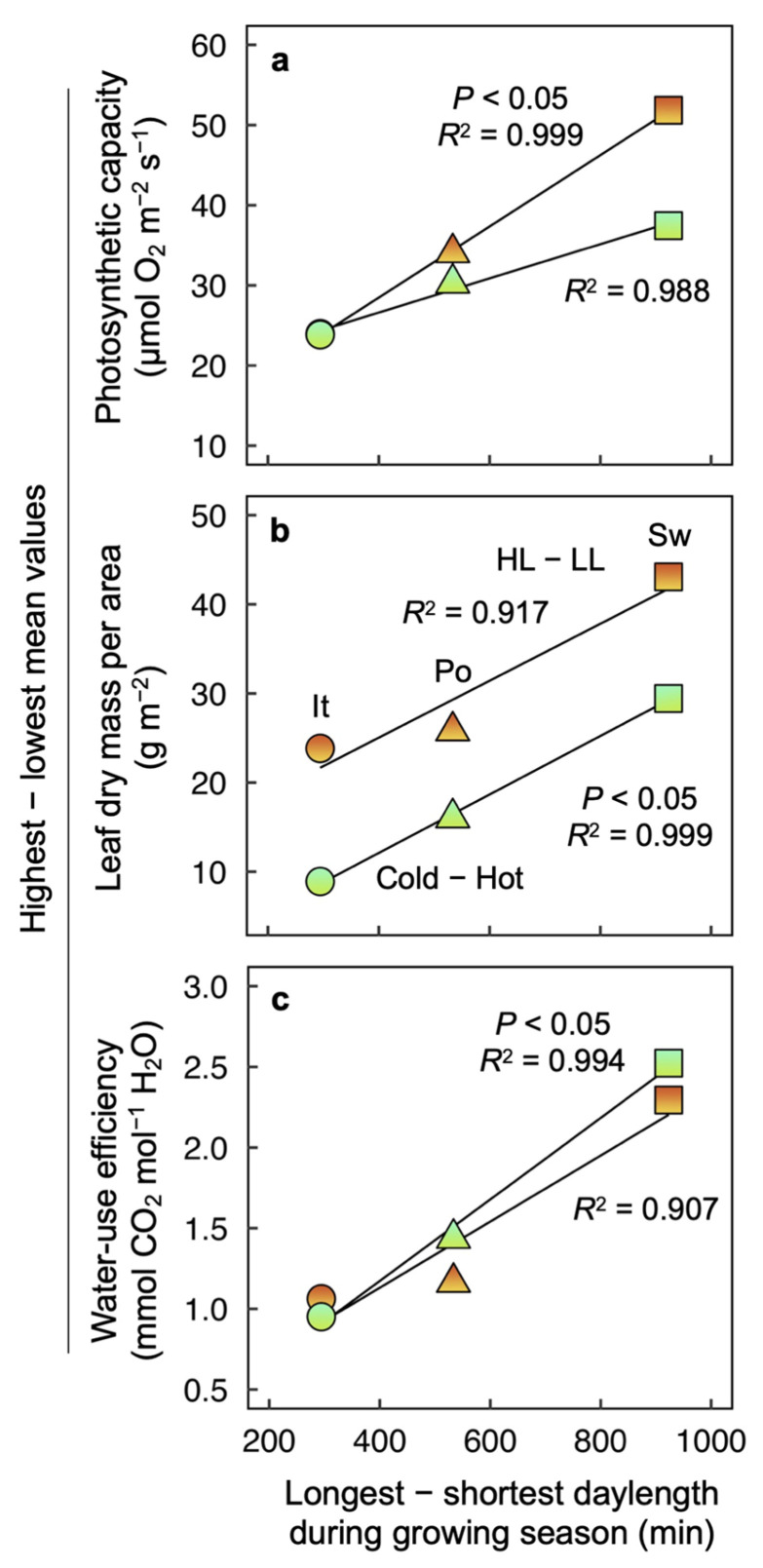
Amplitude of response to growth PFD (light orange to orange symbols) and growth temperature (olive-green to blue-green symbols) as a function of variation in daylength at the site or origin for (**a**) light- and CO_2_-saturated rate of photosynthetic oxygen evolution determined at 25 °C, (**b**) leaf dry mass per area, and (**c**) water-use efficiency in leaves of *Arabidopsis thaliana* ecotypes from Italy (It, circles), Poland (Po, triangles), and Sweden (Sw, squares) that developed under a 9-h photoperiod. The amplitude of response to growth PFD was calculated as the differences in response between plants grown under 1000 and 100 µmol photons m^−2^ s^−1^ at a leaf temperature of 20° (HL–LL), and the amplitude of response to growth temperature was calculated as the difference in response between plants grown at a leaf temperature of 14 °C and 36 °C under 400 µmol photons m−^2^ s^−1^ (Cold—Hot). See legends of Figure 1, Figure 5, Figure 6 and Figure 7 and Materials and Methods for additional information.

Figure 8 features the calculated difference for three traits in plants grown under high versus low PFD (from Figure 5) or grown under low versus high temperature (Figure 6 and Figure 7) as a function of the range of daylengths (longest–shortest) in the sites of ecotype origin. This daylength range was a similarly good predictor of the response to experimental growth PFD or growth temperature of the traits of photosynthetic capacity (Figure 8a), leaf mass (over three-fold difference between Italian and Swedish ecotypes grown at the two temperatures; Figure 8b), and water-use efficiency (two-fold difference in ratio of instantaneous CO_2_-uptake rate to transpirational water loss in the Swedish versus Italian ecotype; Figure 8c). The fact that the difference in leaf mass in high versus low PFD was consistently greater than the difference in cold versus hot temperature resulting from a very low leaf mass in low PFD (Figure 5b).

### 2.3. Interrelationships among Leaf Functional, Morphological, and Multiple Vascular Features

**Figure 9:**
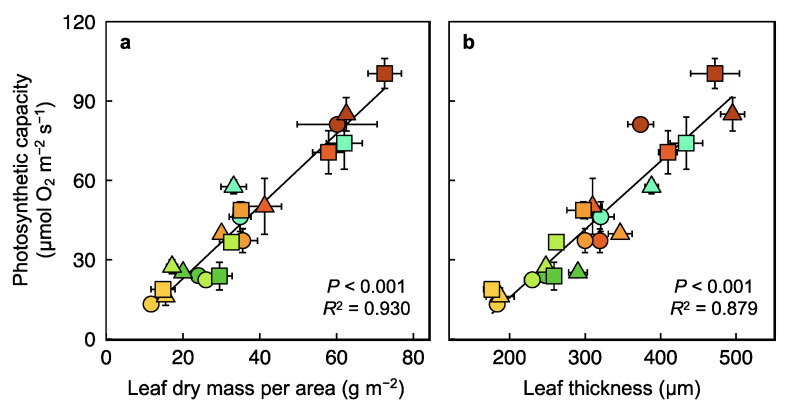
Relationships between the light- and CO_2_-saturated rate of photosynthetic oxygen evolution determined at 25 °C and (**a**) leaf dry mass per area or (**b**) leaf thickness in leaves of *Arabidopsis thaliana* ecotypes from Italy (circles), Poland (triangles), and Sweden (squares) that developed under a 9-h photoperiod of seven different PFD and temperature regimes. Mean values ± standard deviations (photosynthetic capacity and leaf dry mass per area) or ± standard errors (leaf thickness). See Figure 1b and its legend for a description of the colors used to designate the seven growth regimes. For additional information, see Materials and Methods. Data from Cohu et al. [23,24], Adams et al. [22,25], and Stewart et al. [26,27,28].

Significant positive and linear relationships existed for all ecotypes across the wide range of phenotypic variation induced here by seven experimental growth environments. Photosynthetic capacity exhibited such relationships with leaf mass (Figure 9a) and leaf thickness (Figure 9b), from lowest values in low light and moderate to high temperature to the highest values in high light and/or low temperature.

**Figure 10:**
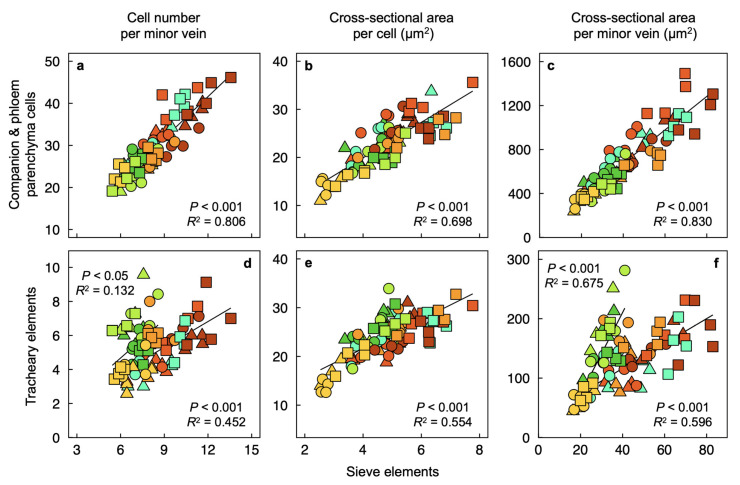
Relationships between (**a**–**c**) companion and phloem parenchyma cells versus sieve elements and (**d**–**f**) tracheary and sieve elements on the bases of (**a**,**d**) cell number per vein, (**b**,**e**) cross-sectional area per cell, and (**c**,**f**) cross-sectional area per foliar minor veins of *Arabidopsis thaliana* ecotypes from Italy (circles), Poland (triangles), and Sweden (squares) that developed under a 9-h photoperiod of seven different PFD and temperature regimes. In panels (**d**) and (**f**), regression lines were analyzed for two separate groups of data: (1) for leaves of plants that developed under low light (yellow symbols), under moderate light and warm temperature (green symbols), and under moderate light at high temperature (olive-green symbols) and (2) excluding leaves of plants that developed under moderate light at warm temperature (green symbols) or high temperature (olive-green symbols). When all data were considered for a single linear regression, *p* < 0.001 for both panels (**d**) and (**f**), and *R^2^* = 0.136 for panel (**d**) and *R^2^* = 0.268 for panel (**f**). See Figure 1b and its legend for a description of the colors used to designate the seven growth regimes. Mean values ± standard errors. For additional information, see Materials and Methods. Data from Cohu et al. [23,24], Adams et al. [22,25], and Stewart et al. [26,27,28].

Foliar minor-vein companion and parenchyma cells of the phloem (both involved in sucrose loading into sieve tubes) correlated closely with sieve elements on several reference bases (Figure 10a–c). These relationships were strongest for the cell number (Figure 10a) and cross-sectional area of the respective cell types (Figure 10c) per minor vein. Notably, relationships between tracheary elements (involved in water transport into the leaf and sugar mass-flow out of the leaf) and sieve elements varied between plants grown in warm-to-hot versus cold conditions when expressed as cell number (Figure 10d) or cross-sectional areas (Figure 10f) per vein but not for cross-sectional area per individual cell (Figure 10e). In particular, plants grown under 400 µmol photons m^−2^ s^−1^ in a warm (green) or hot (olive-green) temperature had high numbers and large resulting total cross-sectional areas per vein of water conduits versus sugar conduits (Figure 10d,f). In contrast, both cold-grown and high-light-grown plants had much lower such ratios of tracheary to sieve elements (Figure 10d,f).

**Figure 11:**
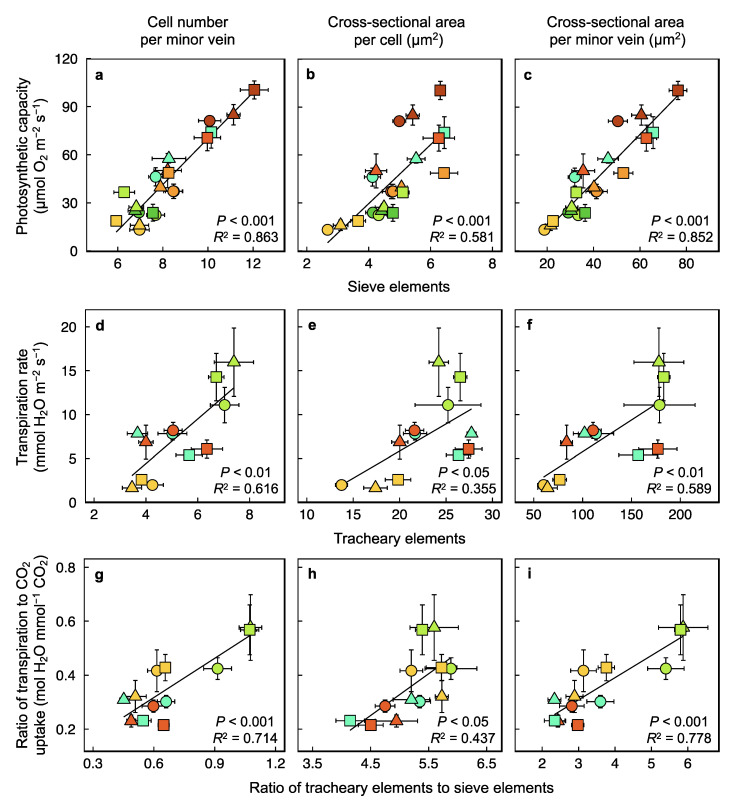
Relationships between (**a**–**c**) sieve elements of foliar minor veins and light- and CO_2_-saturated rate of photosynthetic oxygen evolution determined at 25 °C, (**d**–**f**) tracheary elements of foliar minor veins and the rate of transpirational water loss, and (**g**–**i**) ratio of tracheary to sieve elements of foliar minor veins and ratio of transpirational water loss to carbon dioxide uptake in leaves of *Arabidopsis thaliana* ecotypes from Italy (circles), Poland (triangles), and Sweden (squares) that developed under a 9-h photoperiod of seven different PFD and temperature regimes. See Figure 1b and its legend for a description of colors used to designate the seven growth regimes and legends of Figure 4 and Figure 6 for details of conditions employed to ascertain rates of transpiration and CO_2_ uptake. Mean values ± standard deviations (gas exchange) or ± standard errors (minor vein vascular features). For additional information, see Materials and Methods. Data from Cohu et al. [23,24], Adams et al. [22,25], and Stewart et al. [26,27,28].

Photosynthetic capacity exhibited highly significant linear relationships with number and total cross-sectional area of sugar-exporting sieve elements per vein (Figure 11a,c) and a slightly less close, albeit still significant, relationship with individual sieve element cross-sectional area (Figure 11b). Unlike for the light-and CO_2_-saturated capacity of photosynthetic oxygen evolution, there is no well-defined way to quantify the maximal capacity of transpiration rate. This may be why the foliar transpiration rate measured under the respective growth conditions was less closely correlated with foliar water conduit features (Figure 11d–f) than photosynthetic capacity was with sugar conduits. However, the somewhat normalizing comparison of the ratio of tracheary to sieve elements (especially for cell numbers (Figure 11g) and cross-sectional areas (Figure 11i) per vein) with the ratio of transpiration to CO_2_ uptake yielded correlation coefficients that were higher than those for relationships between transpiration rate and tracheary elements (Figure 11d–f).

## 3. Discussion

### 3.1. Extent of Phenotypic Acclimation to Light and Temperature Mirrors Environmental Variability in Each Ecotype’s Habitat

*Arabidopsis thaliana,* an opportunistic ephemeral, can thrive in spatially and temporally similar microenvironments in different geographic regions. For example, springtime bolting and flowering are shifted such that prevailing temperatures during these events are similar in the Italian (March and April), Polish (April and May), and Swedish (May and June) sites of ecotype origin [22]. Such effects could potentially minimize differences in productivity-related processes among ecotypes grown under common experimental conditions. For plants grown under moderate light and temperature, there was indeed no significant difference in leaf structural features, arrangement of foliar vasculature, and photosynthetic capacity among the three ecotypes. However, leaves grown under extremes of temperature or PFD did exhibit clear differences in the degree of plasticity of the characterized traits. Although outside the scope of this study, underlying genotypic differences among the three ecotypes involve the C-repeat binding factor (CBF) transcription factor family and its target genes as well as other regulators [31,33,56]. Considerable attention in studies of the ecotypic pair from Sweden and Italy has been paid to differences in freezing tolerance [29,30,31,32,33]. We here instead focus on productivity-related traits with an emphasis on the relationship between maximal photosynthetic capacity and the apparent structural capacity for sugar and water movement at the level of individual vascular cells, individual minor veins, and the whole leaf.

The current findings indicate that the magnitude of plant response to experimental variation in growth conditions varied as a function of the magnitude of variation in abiotic features over the growing season in the sites of ecotype origin. Especially for photosynthesis-associated traits, the magnitude of this phenotypic plasticity followed latitudinal gradients in the extent of variation in temperatures and daylengths over the growing season among habitats of ecotype origin, while the extent of phenotypic plasticity in water-transport-related traits was correlated with precipitation level. It is likely that the identification of these relationships for photosynthesis-associated traits was aided by several procedural factors, including the use of extremes in experimental growth PFDs and temperatures. In addition, limitations to sink strength and plant growth were minimized using large rooting volumes [53] and ample nutrient and water supply. Moreover, using the maximal capacity of photosynthesis (rather than the photosynthesis rate under growth conditions) allowed comparison with anatomical features that presumably represent the capacity for the transport of sugars [23,24,57]. Lastly, using fully expanded leaves of plants grown under different conditions from seedling evidently allowed the manifestation of the full extent of acclimation. For example, leaves of the Swedish ecotype grown under a cold leaf temperature of 14 °C exhibited considerably greater upregulation of photosynthetic capacity than leaves grown to maturity under 25 °C and then transferred to 14 °C for just one week [53]. The findings of the present overview thus support the importance of trait choice for characterization, experimental conditions, and environmental features of the site from which the ecotype originated (see also [11]).

Greater plasticity of certain traits in ecotypes from habitats that experience greater environmental variability was also observed in other studies [2,58,59,60,61,62,63]. For example, Molina-Motenegro and Naya [64] found that, among five different populations of dandelions growing along the west coast of South America (from nearly 0° to over 53° S), the phenotypic plasticity of several traits (including photosynthesis and water use efficiency) in response to two growth temperatures was greatest in populations from higher latitudes and least in populations from lower latitudes, which is similar to the present findings for photosynthesis, water use efficiency, leaf mass and thickness, and minor vein phloem features.

It should be noted that only a select few traits were evaluated in the present study, even when just considering foliar vascular traits. Additional traits, such as transfer cell wall ingrowths in minor vein phloem cells, exhibited increases in response to development under both higher light intensity [41,65] and lower temperature [66], resulting in increased cell-membrane invagination and area available for the placement of proteins (sucrose efflux transporters and ATPases), which could contribute to greater levels of phloem loading in *A. thaliana* [67]. Such invaginations are also part of the suite of acclimatory responses that exhibit variation along the latitudinal gradient in temperature and daylength among the three ecotypes characterized here [22,25,66]. However, not all traits respond in the same direction among populations sampled across a transect of differing climates, as discussed further in the next subsection.

### 3.2. The Magnitude of Phenotypic Plasticity Varies by Trait

As detailed here, the Swedish ecotype exhibited more pronounced upregulation of maximal photosynthetic capacity and foliar sugar-export capacity than the Italian ecotype when grown under cold temperature and/or high PFD. This response allows the Swedish ecotype to compensate more fully for the depression of activity of photosynthetic enzymes, as well as, presumably, that of membrane-spanning proteins involved in sugar loading under cold temperatures [24,53]. This response may permit the Swedish ecotype to maintain photosynthetic productivity in its native environment with extended periods of cold temperatures over the growing season. In contrast, the less pronounced upregulation of maximal photosynthetic capacity and foliar sugar-export capacity in the Italian ecotype was associated with a more pronounced adjustment of various processes that prevent oxidative damage from excess excitation energy when the utilization of absorbed light through photosynthesis is diminished. In particular, the Italian ecotype downregulated genes of light-harvesting complexes more than the Swedish ecotype under cold temperatures and high light [56], which apparently helped diminish excitation pressure [33]. In addition, the Italian ecotype upregulated multiple antioxidation processes that counteract oxidative stress more strongly than the Swedish ecotype [56]. These responses may allow the Italian ecotype to persist throughout periods of cold temperature in a state well protected against oxidative stress despite diminished photosynthetic productivity. In other words, the lesser magnitude of phenotypic plasticity in traits associated with photosynthetic productivity under cold temperature and high light is thus accompanied by a greater magnitude of phenotypic plasticity in processes that offer protection when photosynthetic productivity is diminished at cold temperature in the Italian ecotype. It should be noted that the Italian ecotype experiences low temperatures to a lesser degree than the Swedish ecotype in the respective native habitats [21,22].

The most pronounced upregulation of the foliar water-transport infrastructure and transpiration rate under experimental growth with warm to hot temperature was seen in the Polish ecotype from the site of origin with the lowest monthly precipitation over the growing season. This finding is consistent with previous reports of a positive association between foliar vein density and aridity across different species [68,69] and, most notably, between higher vein density and lower precipitation among seven species of the herbaceous genus *Plantago* [70].

### 3.3. Relationships between Photosynthesis and Leaf Structure as Well as among Multiple Leaf Vascular Features

Several common relationships held over the large range of values in various parameters (from three- to four-fold among the anatomical features and over seven- to eight-fold for gas exchange) generated here by growing several ecotypes under multiple combinations of growth conditions, including extremes in light and temperature. Positive associations between photosynthetic capacity and both leaf mass and thickness are typically found among leaves acclimated to different temperatures and PFDs [25,38,39,40,41,42,43,44,45,46,47,48,49,50,51]) and among different cultivars [71,72]. In a meta-analysis of 760 species, leaf dry mass per area, leaf thickness, and photosynthesis were all found to increase with increasing daily receipt of light [73]. Carriquí et al. [74] documented a similar response to three PFDs (from 75 to 490 µmol photons m^−2^ s^−1^) for the Polish ecotype of *A. thaliana*. For plants in the present analysis, greater leaf thickness was due largely to acclimatory plasticity in the number of palisade mesophyll cells, from two layers in low PFD and higher temperature to up to five layers in high PFD and low temperature ([25,50]; see also [75]). Such additional mesophyll cells presumably accommodate more chloroplasts per unit leaf area [41].

On the other hand, a weaker relationship or no relationship between photosynthesis and either leaf thickness or dry mass per area was reported for some woody species ([76,77]; see also [78] and, for ferns, [79]). This may be due to considerable secondary cell-wall formation in sclerophytic leaves, the dry mass of which does not contribute to photosynthesis. One notable exception reported by Niinemets [80] was a positive correlation between the light-saturated photosynthesis rate under ambient CO_2_ with leaf dry mass per area in leaves of two subspecies of the oak *Quercus ilex* across southern Europe. Consistent with our findings, winter-deciduous silver birch from northern Finland had a higher photosynthesis rate compared to a southern population when both were grown in pots (1.5 L) at 20 °C under a 20-h photoperiod of 150 to 600 µmol photons m^−2^ s^−1^ [81]. On the other hand, leaf thickness and photosynthesis were greater in populations of summer-active (winter-deciduous) *Juglans regia* (walnut) from lower compared to higher latitudes among 11 accessions grafted onto rootstocks in pots and grown in a lathe house during hot summer months in Davis, California ([72]; see [82] for a similar finding in deciduous oak species growing in Gipuzkoa, Spain). These results may be comparable to the absence of significant differences in leaf thickness and photosynthesis among *A. thaliana* ecotypes grown under the warm temperature and moderate PFD reported here.

The finding that the strongest relationships between foliar vascular parameters and gas exchange were those between phloem features of minor veins and maximal photosynthetic capacity across all experimental conditions used here is consistent with previous assessments of winter annuals [24,50,83]. Such coordination should ensure that the capacity for the export of sugars produced in photosynthesis matches the capacity for producing such a photosynthate. Concerning significant correlations among individual vascular cell types, such coordination should be expected for sugar-exporting sieve elements that cooperate not only with companion and phloem parenchyma cells involved in sugar loading but also with tracheary elements that have a role in supporting sugar mass flow out of the leaf [55].

Another fraction of water transported by foliar water conduits is lost through stomata as CO_2_ enters the leaf, and this transpirational water loss relative to CO_2_ intake varies with temperature. This variable association depends on both the ecotype and the experimental growth environment. The trend seen here for higher levels of transpiration and lower photosynthetic capacities in high-light-grown leaves of the Italian versus the Swedish ecotype may indicate a genetic propensity for greater transpirational water loss in the Italian ecotype from the lower latitude with a warmer climate. In addition, leaves of all ecotypes grown under warm-to-hot temperatures exhibited greater numbers and cross-sectional areas per vein for tracheary versus sieve elements. Together, these effects presumably allow for greater water delivery in support of higher transpirational water loss at high temperatures in warm-grown leaves, particularly in the ecotype from the lower latitude with a warmer climate.

### 3.4. Recommendations for Future Research

More research is needed to define the range and type of experimental growth conditions that allow for the assessment of the phenotypic plasticity of traits or suites of traits as well as suitable numbers and sites of origin of ecotypes for comparison. A wide range of experimental growth conditions, the use of mature plants, and the minimization of sink limitation appeared to be advantageous for the investigation of foliar parameters related to plant productivity. In the present study, the use of relatively large plants (light-exposed leaf areas of approximately 100 cm^2^ [26,27,84] and diameters of approximately 10 cm [33]) grown to maturity in large pots under contrasting growth conditions resulted in very thick leaves and high photosynthetic capacities under high-growth PFDs and lower temperatures. Future studies are needed to assess whether sink limitation affects relationships measured in smaller, younger plants growing in smaller pots (see Carriquí et al. [74], in which *A. thaliana* plants were grown in 200 mL of soil, personal communication M. Carriquí). The PFD at which *A. thaliana* is typically grown experimentally ranges between 90 and 160 µmol photons m^−2^ s^−1^ (see recommendations in https://abrc.osu.edu/educators/growing [accessed on 18 May 2023] and https://www.arabidopsis.org/download_files/Protocols/abrc_plant_growth.pdf [accessed on 18 May 2023]; e.g., [85,86,87,88,89,90,91]) with growth temperatures around 22 °C. Could it be that these conditions were deemed optimal due to restrictions on root growth in Petri plates, small pots, or shallow flats, which may place restrictions on shoot growth (Figure 2) and the extent of upregulation of photosynthesis and associated traits? Such conditions may thus impact the nature of relationships found in comparisons of ecotypes. As a weedy species that colonizes disturbed sites and thrives in open and rocky habitats [92], *A. thaliana* should be expected to be tolerant of full sunlight, which is up to 20 times greater than the PFD typically deemed optimal for this species. The characterization of 30 *A. thaliana* ecotypes across a gradient from central Sweden to the southwestern Iberian Peninsula (i.e., a latitudinal and longitudinal gradient) grown for 14 weeks in 130 mL pots under low versus high temperature and two watering regimes revealed greater plasticity for most characterized traits among the ecotypes from the center compared to extremes of the gradient [93]. This outcome is similar to the finding of high phenotypic plasticity of water-related traits (transpiration, vein density, xylem features) of the Polish ecotype in the present study but differs from the conclusion drawn here with respect to the exceptionally high phenotypic plasticity of photosynthesis-related traits in the Swedish ecotype from high latitude. In another study involving 15 *A. thaliana* ecotypes grown in a greenhouse with a mean temperature of 23 °C and a PFD of 193 µmol photons m^−2^ s^−1^ (i.e., similar to the moderate conditions of the current study where no differences among the three ecotypes were found), the growth rate was unrelated to the environmental features of the sites of origin [94]. Future research is needed to elucidate to what extent such different findings may be due to the specific traits considered or due to plant growth conditions.

Another question is whether differences in phenotypic plasticity characterized here have an impact on fitness [6,11,36,95]. For example, it remains to be determined whether fitness is affected by acclimatory adjustments in foliar structural and functional traits or rather depends on other aspects of plant structure and function associated, e.g., with the root system, the process of bolting and flowering, or aspects of phenology [11,61,95]. Furthermore, because *A. thaliana* has mesophytic leaves with a high photosynthetic capacity, as well as a short lifespan, the insight gained from such a species should not necessarily be expected to apply to species with long-lived sclerophytic leaves and a low maximal photosynthetic capacity (see [36]). In particular, the relationships revealed here may be typical for a species such *A. thaliana* that is responsive to not only growth light environment but also especially to temperature (as a winter annual). Summer annuals (and some biennials) have been reported to exhibit little phenotypic plasticity of some of the traits characterized here in response to temperature (see, e.g., [50,96]).

### 3.5. Summary of Major Findings

*Arabidopsis thaliana* ecotypes from native habitats with more pronounced differences in daylength or temperature, or with lower precipitation, exhibited more pronounced foliar acclimatory adjustments in response to contrasting experimental light or temperature conditions during leaf development. The extent of the phenotypic plasticity of features associated with photosynthesis, such as water-use efficiency of CO_2_ uptake, leaf mass and thickness, and foliar sugar-transport infrastructure, was predicted by the extent of variability in the daylength and temperature of the native habitats along a latitudinal gradient. On the other hand, the extent of phenotypic plasticity in he transpiration rate and water-transport infrastructure (vein density, ratio of water to sugar conduits) was predicted by the precipitation experienced by each ecotype during its respective growing season, which did not correspond to a latitudinal gradient. These findings indicate that the phenotypic plasticity of various photosynthesis-associated foliar features can vary independently and as predicted by different specific features of the native habitat.

Moreover, the use of seven experimental growth regimes with different combinations of temperature and PFD revealed fundamental structural and functional relationships across conditions for all three ecotypes. These common relationships included significant positive, linear correlations between (1) the leaf mass or thickness and the photosynthetic capacity; (2) the photosynthate-export infrastructure (features of minor-vein sieve elements) and the photosynthetic capacity; (3) the water-transport infrastructure (features of minor-vein tracheary elements) and the transpiration rate; (4) the ratio of tracheary-to-sieve elements (both number and cross-sectional area per foliar minor vein) and the ratio of transpiration-to-CO_2_ uptake; (5) the sugar-loading companion and phloem-parenchyma cells and sugar-exporting sieve elements (both number and cross-sectional area per foliar minor vein, as well as cross-sectional area of individual cells); and (6) the tracheary elements and sieve elements (both number and cross-sectional area per minor vein, as well as cross-sectional area of individual cells). Such coordination among multiple structural and functional foliar features presumably maintains competent execution of function during profound acclimatory adjustment of leaves to different and changing environmental conditions.

## 4. Materials and Methods

### 4.1. Plant Material, Climatological Information, and Growth Conditions

Three ecotypes of *Arabidopsis thaliana* (L.) Heynhold originating in Sweden, Poland, and Italy (Figure 1a) were grown under seven different temperature and PFD regimes (Figure 1b). The Polish ecotype (Columbia-0) was obtained from *The Arabidopsis Information Resource* (https://www.arabidopsis.org [accessed on 18 May 2023]), and a description of the Swedish and Italian ecotypes can be found in Stewart et al. [84]. Individual plants were grown from seed, each in 2.9 L of Canadian Growing Mix 2 (Conrad Fafard Inc., Agawam, MA, USA), in E-15 or PGR15 growth chambers (Conviron, Winnipeg, MB, Canada), except for plants grown in 50 mL of Canadian Growing Mix 2 shown in Figure 2. For additional detail regarding germination and establishment under different combinations of temperature and PFD, see Cohu et al. [23], Adams et al. [22], and Stewart et al. [26,27,28]. A detailed description of climate for each site of origin and information on the duration of the growing season (periods of autumn germination and spring reproduction) for each ecotype are presented in Adams et al. [22]. Differences in the daylength during the growing season in each habitat of origin are presented in Figure 3a, differences in temperature during the growing season in each habitat of origin are presented in Figure 3b, and the average monthly precipitation during the growing season in the habitat from which each ecotype originated is presented in Figure 3c.

### 4.2. Leaf Metrics

All foliar parameters were obtained from leaves that developed and expanded fully under each combination of growth temperature and PFD. For the present analysis, these parameters included (1) mm minor vein (third- and fourth-order veins) length per mm^2^ leaf area (vein density) from leaf tissue that had been cleared; (2) numbers and (3) cross-sectional areas of xylem and phloem cells per minor vein; as (4) leaf thickness from tissue that was fixed, embedded, and sectioned for light microscopy; (5) leaf dry mass per unit leaf area; (6) photosynthetic capacity as the light- and CO_2_-saturated rate of oxygen evolution in a water-saturated atmosphere from leaf discs at 25 °C (Hansatech oxygen electrode systems, King’s Lynn, Norfold, UK); and the (7) rates of transpirational water loss and CO_2_ uptake (LCi Portable Photosynthesis System, ADC Bioscientific Ltd., Hoddesdon, Herts, England, UK). For additional details, see figure legends and Cohu et al. [23,24], Adams et al. [22,25], and Stewart et al. [26,27,28,57], from which all of the original foliar data presented in this meta-analysis can be found, with the exception of some previously unpublished leaf dry mass per unit area values. Mean values ± standard deviation are displayed for vein density, photosynthetic capacity, transpiration rate, and leaf dry mass per unit leaf area (*n* = 3 to 5, e.g., one measured leaf section from each of three to five different plants) and mean values ± standard error are displayed for leaf thickness, phloem cells per minor vein, sieve element cross-sectional area per minor vein, minor vein ratio of tracheary to sieve elements, and minor vein ratio of tracheary element cross-sectional area to sieve element cross-sectional area (*n* = 3 to 5, i.e., mean values from three leaf cross-sections or 7–10 minor vein cross-sections within a leaf section from each of three to five plants; for a detailed description of these measurements, see [57]).

### 4.3. Data Analyses

Data were analyzed and visualized with R 4.2.2 (https://www.r-project.org/ [accessed on 18 May 2023]). Bivariate relationships were evaluated via linear regression with the *lm* function, and differences between multiple means were evaluated via one-way ANOVA and post hoc Tukey–Kramer HSD tests with the *aov* and *TukeyHSD* functions, respectively. Data were visualized with the *ggplot2* package [97].

## Data Availability

All data are reported directly in this paper.

## References

[1] Sultan S.E. (2000). Phenotypic plasticity for plant development, function and life history. Trends Plant Sci..

[2] Schlichting C.D., Smith H. (2002). Phenotypic plasticity: Linking molecular mechanisms with evolutionary outcomes. Evol. Ecol..

[3] Donohue K. (2003). Setting the stage: Phenotypic plasticity as habitat selection. Int. J. Plant Sci..

[4] Donohue K. (2013). Development in the wild: Phenotypic plasticity. Annu. Plant Rev..

[5] De Kroon H., Huber H., Stuefer J.F., Van Groenendael J.M. (2005). A modular concept of phenotypic plasticity in plants. New Phytol..

[6] Van Kleunen M., Fischer M. (2005). Constraints on the evolution of adaptive phenotypic plasticity in plants. New Phytol..

[7] Atkin O.K., Loveys B.R., Atkinson L.J., Pons T.L. (2006). Phenotypic plasticity and growth temperature: Understanding interspecific variability. J. Exp. Bot..

[8] Valladares F., Gianoli E., Gómez J.M. (2007). Ecological limits to plant phenotypic plasticity. New Phytol..

[9] Matesanz S., Ramírez-Valiente J.A. (2019). A review and meta-analysis of intraspecific differences in phenotypic plasticity: Implication to forecast plant responses to climate change. Glob. Ecol. Biogeogr..

[10] Stotz G.C., Salgado-Luarte C., Escobedo V.M., Valladares F., Gianoli E. (2021). Global trends in phenotypic plasticity. Ecol. Lett..

[11] Schneider H.M. (2022). Characterization, costs, cues and future perspectives of phenotypic plasticity. Ann. Bot..

[12] Niklas K.J. (2009). Functional adaptation and phenotypic plasticity at the cellular and whole plant level. J. Biosci..

[13] Yu P., White P.J., Hochholdinger F., Li C. (2014). Phenotypic plasticity of the maize root system in response to heterogeneous nitrogen availability. Planta.

[14] Markesteijn L., Poorter L., Bongers F. (2007). Light-dependent leaf trait variation in 43 tropical dry forest tree species. Am. J. Bot..

[15] Legner N., Fleck S., Leuschner C. (2013). Low light acclimation in five temperate broad-leaved tree species of different successional status: The significance of a shade canopy. Ann. For. Sci..

[16] Legner N., Fleck S., Leuschner C. (2014). Within-canopy variation in photosynthetic capacity, SLA and foliar N in temperate broad-leaved trees with contrasting shade tolerance. Trees.

[17] Dos Santos J., Marenco R.A., Ferreira W.C., Dias D.P. (2021). Leaflet phenotypic plasticity in three woody species in two strata of a gallery forest. CERNE.

[18] Rooney R., Ishii H.R., Cavaleri M.A. (2022). Intra-crown variation of leaf mass per area of *Fagus crenata* is driven by light acclimation of leaf thickness and hydraulic acclimation of leaf density. Ecol. Res..

[19] Griffith C., Eunsuk K., Donohue K. (2004). Life-history variation and adaptation in the historically mobile plant *Arabidopsis thaliana* (Brassicaceae) in North America. Am. J. Bot..

[20] Rutter M.T., Fenster C.B. (2007). Testing for adaptation to climate in *Arabidopsis thaliana*: A calibrated common garden approach. Ann. Bot..

[21] Ågren J., Schemske D.W. (2012). Reciprocal transplants demonstrate strong adaptive differentiation of the model organism *Arabidopsis thaliana* in its native range. New Phytol..

[22] Adams W.W., Stewart J.J., Cohu C.M., Muller O., Demmig-Adams B. (2016). Habitat temperature and precipitation of *Arabidopsis thaliana* ecotypes determine the response of foliar vasculature, photosynthesis, and transpiration to growth temperature. Front. Plant Sci..

[23] Cohu C.M., Muller O., Demmig-Adams B., Adams W.W. (2013). Minor loading vein acclimation for three *Arabidopsis thaliana* ecotypes in response to growth under different temperature and light regimes. Front. Plant Sci..

[24] Cohu C.M., Muller O., Stewart J.J., Demmig-Adams B., Adams W.W. (2013). Association between minor loading vein architecture and light- and CO_2_-saturated photosynthetic oxygen evolution among *Arabidopsis thaliana* ecotypes from different latitudes. Front. Plant Sci..

[25] Adams W.W., Stewart J.J., Polutchko S.K., Demmig-Adams B., Adams W.W., Terashima I. (2018). Leaf vasculature and the upper limit of photosynthesis. The Leaf: A Platform for Performing Photosynthesis. Advances in Photosynthesis and Respiration.

[26] Stewart J.J., Demmig-Adams B., Cohu C.M., Wenzl C.A., Muller O., Adams W.W. (2016). Growth temperature impact on leaf form and function in *Arabidopsis thaliana* ecotypes from northern and southern Europe. Plant Cell Environ..

[27] Stewart J.J., Polutchko S.K., Adams W.W., Demmig-Adams B. (2017). Acclimation of Swedish and Italian ecotypes of *Arabidopsis thaliana* to light intensity. Photosynth. Res..

[28] Stewart J.J., Polutchko S.K., Adams W.W., Cohu C.M., Wenzl C.A., Demmig-Adams B. (2017). Light, temperature and tocopherol status influence foliar vascular anatomy and leaf function in *Arabidopsis thaliana*. Physiol. Plant..

[29] Oakley C.G., Ågren J., Atchison R.A., Schemske D.W. (2014). QTL mapping of freezing tolerance: Links to fitness and adaptive trade-offs. Mol. Ecol..

[30] Gehan M.A., Park S., Gilmour S.J., An C., Lee C.-M., Thomashow M.F. (2015). Natural variation in the C-repeat binding factor cold response pathway correlates with local adaptation of Arabidopsis ecotypes. Plant J..

[31] Park S., Gilmour S.J., Grumet R., Thomashow M.F. (2018). CBF-dependent and CBF-independent regulatory pathways contribute to the differences in freezing tolerance and cold-regulated gene expression of two Arabidopsis ecotypes locally adapted to sites in Sweden and Italy. PLoS ONE.

[32] Sanderson B.J., Park S., Jameel M.I., Kraft J.C., Thomashow M.F., Schemske D.W., Oakley C.G. (2020). Genetic and physiological mechanisms of freezing tolerance in locally adapted populations of a winter annual. Am. J. Bot..

[33] Baker C.R., Stewart J.J., Amstutz C.L., Johnson J.D., Ching L.G., Niyogi K.K., Adams W.W., Demmig-Adams B. (2022). Genotype-dependent contribution of CBF transcription factors to long-term acclimation to high light and cool temperature. Plant Cell Environ..

[34] Adams W.W., Adams W.W., Terashima I. (2018). Preface: The importance of leaves to life and humanity. The Leaf: A Platform for Performing Photosynthesis. Advances in Photosynthesis and Respiration.

[35] Adams W.W., Terashima I. (2018). The Leaf: A Platform for Performing Photosynthesis. Advances in Photosynthesis and Respiration.

[36] Niinemets Ü. (2020). Leaf trait plasticity and evolution in different plant functional types. Annu. Plant Rev..

[37] Anderegg L.D.L. (2023). Why can’t we predict traits from the environment?. New Phytol..

[38] Givnish T.J. (1988). Adaptation to sun and shade: A whole-plant perspective. Aust. J. Plant Physiol..

[39] Boese S.R., Huner N.P.A. (1990). Effect of growth temperature and temperature shifts on spinach leaf morphology and photosynthesis. Plant Physiol..

[40] Terashima I., Miyazawa S.I., Hanba Y. (2001). Why are sun leaves thicker than shade leaves?—Consideration based on analyses of CO_2_ diffusion in the leaf. J. Plant Res..

[41] Amiard V., Mueh K.E., Demmig-Adams B., Ebbert V., Turgeon R., Adams W.W. (2005). Anatomical and photosynthetic acclimation to the light environment in species with differing mechanisms of phloem loading. Proc. Natl. Acad. Sci. USA.

[42] Li Z., Zhang S., Hu H., Li D. (2008). Photosynthetic performance along a light gradient as related to leaf characteristics of a naturally occurring *Cypripedium flavum*. J. Plant Res..

[43] Poorter H., Niinemets Ü., Poorter L., Wright I.J., Villar R. (2009). Causes and consequences of variation in leaf mass per area (LMA): A meta-analysis. New Phytol..

[44] Gorsuch P.A., Pandey S., Atkin O.K. (2010). Temporal heterogeneity of cold acclimation phenotypes in *Arabidopsis* leaves. Plant Cell Environ..

[45] Zhou S.B., Liu K., Zhang D., Li Q.F., Zhu G.P. (2010). Photosynthetic performance of *Lycoris radiata* var. *radiata* to shade treatments. Photosynthetica.

[46] Dumlao M.R., Darehshouri A., Cohu C.M., Muller O., Mathias J., Adams W.W., Demmig-Adams B. (2012). Low temperature acclimation of photosynthetic capacity and leaf morphology in the context of phloem loading type. Photosynth. Res..

[47] Tosens T., Niinemets Ü., Vislap V., Eichelmann H., Díez P.C. (2012). Developmental changes in mesophyll diffusion conductance and photosynthetic capacity under different light and water availabilities in *Populus tremula*: How structure constrains function. Plant Cell Environ..

[48] Zhang S.B., Yin L.X. (2012). Plasticity in photosynthesis and functional leaf traits of *Meconopsis horridula* var *racemosa* in response to irradiance. Bot. Stud..

[49] Cai Y.-F., Li S.-F., Li S.-F., Xie W.-J., Song J. (2014). How do leaf anatomies and photosynthesis of three *Rhododendron* species relate to their natural environments?. Bot. Stud..

[50] Cohu C.M., Muller O., Adams W.W., Demmig-Adams B. (2014). Leaf anatomical and photosynthetic acclimation to cool temperature and high light in two winter versus two summer annuals. Physiol. Plant..

[51] Muller O., Cohu C.M., Stewart J.J., Protheroe J.A., Demmig-Adams B., Adams W.W. (2014). Association between photosynthesis and contrasting features of minor veins in leaves of summer annuals loading phloem via symplastic versus apoplastic routes. Physiol. Plant..

[52] Strand A., Hurry V., Henkes S., Huner N., Gustafsson P., Gardeström P., Stitt M. (1999). Acclimation of Arabidopsis leaves developing at low temperatures. Increasing cytoplasmic volume accompanies increased activities of enzymes in the Calvin Cycle and in the sucrose-biosynthesis pathway. Plant Physiol..

[53] Adams W.W., Stewart J.J., Demmig-Adams B., Adams W.W., Terashima I. (2018). Photosynthetic modulation in response to plant activity and environment. The Leaf: A Platform for Performing Photosynthesis. Advances in Photosynthesis and Respiration.

[54] Nagel K.A., Putz A., Gilmer F., Heinz K., Fischbach A., Pfeifer J., Faget M., Blossfeld S., Ernst M., Dimaki C. (2012). GROSCREEN-Rhizo is a novel phenotyping robot enabling simultaneous measurement of root and shoot growth for plants grown in soil-filled rhizotrons. Funct. Plant Biol..

[55] Adams W.W., Stewart J.J., Polutchko S.K., Demmig-Adams B. (2022). Foliar sieve elements: Nexus of the leaf. J. Plant Physiol..

[56] Demmig-Adams B., Polutchko S.K., Baker C.R., Stewart J.J., Adams W.W. (2022). Distinct cold acclimation of productivity traits in *Arabidopsis thaliana* ecotypes. Int. J. Mol. Sci..

[57] Stewart J.J., Muller O., Cohu C.M., Demmig-Adams B., Adams W.W., Liesche J. (2019). Quantification of foliar phloem infrastructure with microscopy. Phloem. Methods and Protocols. Methods in Molecular Biology.

[58] Alpert P., Simms E.L. (2002). The relative advantages of plasticity and fixity in different environments: When is it good for a plant to adjust?. Evol. Ecol..

[59] Gianoli E. (2004). Plasticity of traits and correlations in two populations of *Convolvulus arvensis* (Convolvulaceae) differing in environmental heterogeneity. Intl. J. Plant Sci..

[60] Gianoli E., González-Teuber M. (2005). Environmental heterogeneity and population differentiation in plasticity to drought in *Convolvulus chilensis* (Convolvulaceae). Evol. Ecol..

[61] Molina-Montenegro M.A., Atala C., Gianoli E. (2010). Phenotypic plasticity and performance of *Taraxacum officinale* (dandelion) in habitats of contrasting environmental heterogeneity. Biol. Invasions.

[62] Carvajal D.E., Loayza A.P., Rios R.S., Gianoli E., Squeo F.A. (2017). Population variation in drought-resistance strategies in a desert shrub along an aridity gradient: Interplay between phenotypic plasticity and ecotypic differentiation. Perspec. Plant Ecol. Evol. Syst..

[63] Sheepens J.F., Deng Y., Bossdorf O. (2018). Phenotypic plasticity in response to temperature fluctuations is genetically variable, and relates to climatic variability of origin, in *Arabidopsis thaliana*. AoB Plants.

[64] Molina-Montenegro M.A., Naya D.E. (2012). Latitudinal patterns in phenotypic plasticity and fitness-related traits: Assessing the climatic variability hypothesis (CVH) with an invasive plant species. PLoS ONE.

[65] Amiard V., Demmig-Adams B., Mueh K.E., Turgeon R., Combs A.F., Adams W.W. (2007). Role of light and jasmonic acid signaling in regulating foliar phloem cell wall ingrowth development. New Phytol..

[66] Adams W.W., Cohu C.M., Amiard V., Demmig-Adams B. (2014). Associations between phloem-cell wall ingrowths in minor veins and maximal photosynthesis rate. Front. Plant Sci..

[67] Duan Z., Homma A., Kobayashi M., Nagata N., Kaneko Y., Fujiki Y., Nishida I. (2014). Photoassimilation, assimilate translocation and plasmodesmal biogenesis in the source leaves of *Arabidopsis thaliana* grown under an increased atmospheric CO_2_ concentration. Plant Cell Physiol..

[68] Uhl D., Mosbrugger V. (1999). Leaf venation density as a climate and environmental proxy: A critical review and new data. Paleogeogr. Paleoclimatol. Paleoecol..

[69] Sack L., Scoffoni C. (2013). Leaf venation: Structure, function, development, evolution, ecology and applications in the past, present and future. New Phytol..

[70] Dunbar-Co S., Sporck M.J., Sack L. (2009). Leaf trait diversification and design in seven rare taxa of the Hawaiian *Plantago* radiation. Int. J. Plant Sci..

[71] Han J.M., Zhang Y.J., Lei Z.Y., Zhang W.F., Zhang Y.L. (2019). The higher area-based photosynthesis in *Gossypium hirsutum* L. is mostly attributed to leaf thickness. Photosynthetica.

[72] Momayyezi M., Rippner D.A., Duong F.V., Raja P.V., Brown P.J., Kluepfel D.A., Earles J.M., Forrestel E.J., Gilbert M.E., McElrone A.J. (2022). Structural and functional leaf diversity lead to variability in photosynthetic capacity across a range of *Juglans regia* genotypes. New Phytol..

[73] Poorter H., Niinemets Ü., Ntagkas N., Siebenkäs A., Mäenpää M., Matsubara S., Pons T.L. (2019). A meta-analysis of plant responses to light intensity for 70 traits ranging from molecules to whole plant performance. New Phytol..

[74] Carriquí M., Nadal M., Flexas J. (2021). Acclimation of mesophyll conductance and anatomy to light during leaf aging in *Arabidopsis thaliana*. Physiol. Plant..

[75] Hoshino R., Yoshida Y., Tsukaya H. (2019). Multiple steps of leaf thickening during sun-leaf formation in Arabidopsis. Plant J..

[76] Kenzo T., Ichie T., Yoneda R., Kitahashi Y., Watanabe Y., Ninomiya I., Koike T. (2004). Interspecific variation of photosynthesis and leaf characteristics in canopy trees of five species of Diterocarpaceae in a tropical rain forest. Tree Physiol..

[77] Hassiotou F., Renton M., Ludwig M., Evans J.R., Veneklaas E.J. (2010). Photosynthesis at an extreme end of the leaf trait spectrum: How does it relate to high leaf dry mass per area and associated parameters?. J. Exp. Bot..

[78] Niinemets Ü. (1999). Components of leaf dry mass per area—Leaf thickness and density—Alter leaf photosynthetic capacity in reverse directions in woody plants. New Phytol..

[79] Tosens T., Nishida K., Gago J., Coopman R.E., Cabrera H.M., Carriquí M., Laanisto L., Morales L., Nada M., Rojas R. (2016). The photosynthetic capacity in 35 ferns and fern allies: Mesophyll CO_2_ diffusion as a key trait. New Phytol..

[80] Niinemets Ü. (2015). Is there a species spectrum within the world-wide leaf economics spectrum? Major variations in leaf functional traits in the Mediterranean sclerophyll *Quercus ilex*. New Phytol..

[81] Tenkanen A., Suprun S., Oksanen E., Keinänen M., Keski-Saari S., Kontunen-Soppela S. (2021). Strategy by latitude? Higher photosynthetic capacity and root mass fraction in northern than southern silver birch (*Betula pendula* Roth) in uniform growing conditions. Tree Physiol..

[82] Sancho-Knapik D., Ecudero A., Mediavilla S., Scoffoni C., Zailaa J., Cavender-Bares J., Álvarez-Arenas T.G., Molins A., Alonso-Forn D., Ferrio J.P. (2021). Deciduous and evergreen oaks show contrasting adaptive responses in leaf mass per area across environment. New Phytol..

[83] Adams W.W., Cohu C.M., Muller O., Demmig-Adams B. (2013). Foliar phloem infrastructure in support of photosynthesis. Front. Plant Sci..

[84] Stewart J.J., Adams W.W., Cohu C.M., Polutchko S.K., Lombardi E.M., Demmig-Adams B. (2015). Differences in light-harvesting, acclimation to growth-light environment, and leaf structural development between Swedish and Italian ecotypes of *Arabidopsis thaliana*. Planta.

[85] Ibañez C., Poeschl Y., Peterson T., Bellstädt J., Denk K., Gogol-Döring A., Quint M., Delker C. (2017). Ambient temperature and genotype differentially affect developmental and phenotypic plasticity in *Arabidopsis thaliana*. BMC Plant Biol..

[86] Kolodziejczak M., Skibior-Blaszczyk R., Janska H. (2018). *m*-AAA complexes are not crucial for the survival of Arabidopsis under optimal growth conditions despite their importance for mitochondrial translation. Plant Cell Physiol..

[87] Duruflé H., Ranocha P., Mbadinga D.L.M., Déjean S., Bonhomme M., San Clemente H., Viudes S., Eljebbawi A., Delorme-Hinoux V., Sáez-Vásquez J. (2019). Phenotypic trait variation as a response to altitude-related constraints in Arabidopsis populations. Front. Plant Sci..

[88] Duruflé H., Ranocha P., Balliau T., Zivy M., Albenne C., Burlat V., Déjean S., Jamet E., Dunand C. (2020). An integrative study showing the adaptation to sub-optimal growth conditions of natural populations of *Arabidopsis thaliana*: A focus on cell wall changes. Cells.

[89] Gawrónski P., Burdiak P., Scharff L.B., Mielecki J., Górecka M., Zaborowska M., Leister D., Waszczak C., Karpínski S. (2020). CIA2 and CIA2-LIKE are required for optimal photosynthesis and stress responses in *Arabidopsis thaliana*. Plant J..

[90] Velitchkova M., Popova A.V., Faik A., Gerganova M., Ivanov A.G. (2020). Low temperature and high light dependent dynamic photoprotective strategies in *Arabidopsis thaliana*. Physiol. Plant..

[91] Serrato A.J., Rojas-González J.A., Torres-Romero D., Vargas P., Sahrawy M. (2021). Thioredoxins *m* are major players in the multifaceted light-adaptive response in *Arabidopsis thaliana*. Plant J..

[92] Mitchell-Olds T., Schmitt J. (2006). Genetic mechanisms and evolutionary significance of natural variation in *Arabidopsis*. Nature.

[93] Estarague A., Vasseur F., Sartori K., Bastias C.C., Cornet D., Rouan L., Beurier G., Exposito-Alonso M., Herbette S., Bresson J. (2022). Into the range: A latitudinal gradient or a center-margins differentiation of ecological strategies in *Arabidopsis thaliana*. Ann. Bot..

[94] Fletcher L.R., Scoffoni C., Farrell C., Buckley T.N., Pellegrini M., Sack L. (2022). Testing the association of relative growth rate and adaptation to climate across natural ecotypes of *Arabidopsis*. New Phytol..

[95] Bonser S.P. (2021). Misinterpreting the adaptive value of phenotypic plasticity in studies on plant adaptation to new and variable environments. Plant Biol..

[96] Muller O., Stewart J.J., Cohu C.M., Polutchko S.K., Demmig-Adams B., Adams W.W. (2014). Leaf architectural, vascular, and photosynthetic acclimation to temperature in two biennials. Physiol. Plant..

[97] Wickham H. (2016). Ggplot2: Elegant Graphics for Data Analysis.

